# Speech-Evoked Cortical Auditory Potentials as Biomarkers of Auditory Maturation in Children with Cochlear Implants

**DOI:** 10.3390/children13020222

**Published:** 2026-02-04

**Authors:** Zeynel Abidin Karatas, Cengiz Durucu

**Affiliations:** 1 Department of Otorhinolaryngology, Duztepe Yasam Hospital, Gaziantep 27300, Turkey; zeynelkaratas23@gmail.com; 2 Department of Otorhinolaryngology, Faculty of Medicine, Gaziantep University, Gaziantep 27310, Turkey

**Keywords:** cortical auditory evoked potentials, P1 latency, cochlear implant, auditory maturation, pediatric audiology, speech stimuli

## Abstract

**Highlights:**

**What are the main findings?**

- P1 latency—a cortical marker of auditory maturation—was significantly longer in cochlear implant (CI) users than in age-matched normal-hearing (NH) children for low- (/m/) and high-frequency (/t/) speech stimuli.
- In NH children, P1 latency shortened with increasing age, while in CI users it decreased with longer implant-use duration, indicating experience-dependent cortical plasticity.
- No significant difference in P1 latency was observed for mid-frequency (/g/) stimuli, suggesting frequency-specific maturation dynamics.

**What is the implication of the main finding?**

- Speech-evoked cortical auditory evoked potentials (CAEPs) provide a reliable, objective biomarker for assessing auditory cortical development and plasticity in pediatric CI users.
- Integrating multi-frequency CAEP testing into peri-operative and rehabilitation protocols can help monitor cortical maturation, optimize implant programming, and guide individualized auditory-verbal therapy.

**Abstract:**

**Objectives:** This study aimed to evaluate auditory cortical maturation in pediatric cochlear implant (CI) users using speech-evoked cortical auditory evoked potentials (CAEPs) and to compare P1 latency responses with age-matched normal-hearing (NH) peers. Secondary objectives included examining the relationship between P1 latency, age, and duration of implant use to assess experience-dependent cortical plasticity. **Materials and Methods:** Seventy children were enrolled, including 40 prelingually deaf CI users and 30 NH controls matched for age and sex. CAEPs were recorded using the HEARLab system with three speech tokens representing low (/m/), mid (/g/), and high (/t/) frequencies, presented at 55 dB SPL in a free-field setup. The P1 component was identified as the first positive deflection between 50 and 150 ms after stimulus onset. Group comparisons were performed using Student’s *t*-test, and correlations between P1 latency, age, and implant-use duration were analyzed using the Pearson correlation test (*p* < 0.05). **Results:** Mean P1 latencies were significantly longer in CI users than in NH peers for the /m/ and /t/ stimuli (*p* = 0.036 and *p* = 0.045, respectively), while no significant difference was found for /g/ (*p* = 0.542). In NH children, P1 latency negatively correlated with age (r = −0.44, *p* < 0.05), indicating maturation-related shortening. Among CI users, longer implant-use duration was associated with shorter P1 latencies across all speech tokens (/m/: r = −0.37; /g/: r = −0.49; /t/: r = −0.43; *p* < 0.05 for all). **Conclusions:** Speech-evoked CAEPs provide a sensitive and objective measure of auditory cortical development in children with cochlear implants. P1 latency reflects both chronological and hearing-age-related maturation, supporting its clinical use as a biomarker for cortical plasticity and rehabilitation progress in pediatric CI care.

## 1. Introduction

Permanent childhood hearing loss disrupts the auditory input that normally sculpts cortical circuits for speech and language, with downstream effects on communication, cognition, and psychosocial development [1,2,3]. For infants and children with bilateral severe-to-profound sensorineural hearing loss who receive limited benefit from acoustic amplification, cochlear implantation (CI) restores access to speech-relevant signals by encoding sound into patterned electrical stimulation of the auditory nerve, enabling auditory pathway activation and language learning during developmentally sensitive windows [4,5,6]. Beyond behavioral outcomes, electrophysiological measures—particularly cortical auditory evoked potentials (CAEPs)—offer an objective lens on central auditory maturation under restored input. Among CAEP components, the P1 peak (a positive deflection typically recorded ~50–150 ms in children) shows robust age-related latency shortening and morphology changes that index the integrity and maturation of thalamo-cortical and primary auditory cortical processing [7,8,9,10,11,12,13].

A large body of work indicates a sensitive period for central auditory development: implantation during early childhood—often cited around ~3.5 years—is associated with more typical CAEP waveforms and faster P1 latency normalization; after ~7 years of auditory deprivation, plasticity appears markedly constrained and cortical responses become atypical or cross-modally reorganized [14,15,16,17,18,19]. While landmark studies first established these principles, recent clinical research continues to validate P1 latency as a practical biomarker: in pediatric CI users, shorter P1 latencies correlate with better language measures, and longitudinal follow-up shows progressive P1 latency reductions over years of effective device use [1,3,10,20,21]. These patterns align with the notion that hearing age and consistent CI wear time (daily hours, cumulative use) are key drivers of cortical normalization, a relationship now being quantified using both acoustically and electrically evoked CAEP paradigms [2,22,23].

Clinically, CAEPs are increasingly used to verify aided audibility, document cortical access to speech cues, and guide intervention. Feasibility studies in infants and children report acceptable test–retest characteristics and clinical utility for confirming cortical detection of speech-like stimuli (e.g., /m/, /g/, /t/) presented in the sound field, including implementations with systems such as HEARLab [24,25,26,27,28]. In CI users, both scalp-recorded and electrically evoked CAEPs (eCAEPs) demonstrate P1/N1/P2 peaks whose latencies and amplitudes reflect auditory pathway engagement; direct-to-implant recordings are emerging as complementary tools and show close correspondence with scalp measures [29,30,31]. Together, these advances position CAEP P1 latency as a translational bridge between device programming and neurodevelopmental outcomes [32,33]. The frequency-specific normative P1 latency intervals established in the present study (80–120 ms for /m/, 95–135 ms for /g/, and 90–130 ms for /t/) provide clinicians with practical reference thresholds for interpreting cortical maturation patterns in pediatric CI users.

Building upon the existing evidence that cortical auditory evoked potentials—particularly the P1 component—serve as reliable indicators of auditory cortical maturation, this study was designed to evaluate cortical development in children with cochlear implants. The primary objective was to quantify P1 latency responses to speech-like stimuli (/m/, /g/, and /t/) using the HEARLab system and to compare these results with age-matched normal-hearing peers. By correlating P1 latency with age, age at implantation, and duration of implant use, the study aims to provide objective electrophysiological insight into auditory pathway maturation and to position institutional outcomes within the broader framework of contemporary pediatric cochlear implantation research.

## 2. Materials and Methods

### 2.1. Study Design and Patient Selection

This retrospective, observational study was conducted in the Department of Otorhinolaryngology at Gaziantep University Faculty of Medicine between January 2006 and January 2012. It included 40 prelingually deaf children who underwent unilateral cochlear implantation and 30 normal-hearing children who volunteered to participate. The study protocol was approved by the Gaziantep University Medical Faculty Ethics Committee (Approval No. 83, date: 19 March 2013) in accordance with the Declaration of Helsinki. Written informed consent was obtained from the parents or legal guardians of all participants before inclusion.

The normal-hearing group consisted of children aged 15–84 months (mean = 58.9 months) who had no neurological, linguistic, or otologic abnormalities. Hearing screening was performed using acoustic immittance and distortion product otoacoustic emission (DPOAE) tests, and only children passing both tests were enrolled.

The cochlear implant (CI) group included children aged 21–82 months (mean = 53.1 months) with prelingual, bilateral, profound sensorineural hearing loss confirmed by auditory brainstem response (ABR) testing. All CI candidates had used bilateral digital hearing aids for at least 3–6 months preoperatively without adequate benefit, and were implanted with a MED-EL Sonata device. Each patient had undergone intraoperative electrically evoked stapedius reflex threshold (ESRT) testing and postoperative cortical auditory evoked potential (CAEP) measurements. Postoperatively, all children were enrolled in structured auditory–verbal therapy and followed at rehabilitation centers.

Children were eligible for inclusion in the cochlear implant (CI) group if they had prelingual bilateral profound sensorineural hearing loss, had completed at least six months of consistent implant use before cortical auditory evoked potential (CAEP) testing, and regularly participated in structured auditory rehabilitation programs. Only patients with complete and accessible postoperative audiological data were included in the analysis. Children were excluded if they had perilingual or postlingual hearing loss, radiologic or clinical evidence of cochlear malformations, brainstem or central auditory pathway abnormalities, or inconsistent implant use. Those who failed to attend routine rehabilitation sessions or had incomplete postoperative performance records were also excluded from the study. Etiologic information was extracted when available; the most commonly documented causes included congenital nonsyndromic hearing loss, presumed genetic etiology, and perinatal factors. However, etiologic data were incomplete for several participants and therefore were not included in the statistical analysis.

CAEP recordings in the normal-hearing (NH) control group were not obtained specifically for research purposes. These children were evaluated in the audiology clinic for reasons such as suspected auditory processing delay, unclear behavioral thresholds, or speech delay, and CAEP testing was part of routine clinical assessment. Therefore, both CI and NH data were retrospectively retrieved from existing clinical records, justifying the retrospective observational case–control design.

### 2.2. Preoperative Audiological and Radiological Assessment

Before implantation, all patients underwent comprehensive otologic and neurodevelopmental evaluation. Audiological testing included tympanometry, acoustic reflexes, pure-tone or free-field audiometry, ABR, and auditory steady-state response (ASSR) assessments. High-resolution temporal bone computed tomography (CT) and inner-ear magnetic resonance imaging (MRI) were used to verify the integrity of the cochlear nerve and cochlear patency. Children were evaluated by child psychiatry and child neurology departments to exclude cognitive or intellectual disability.

### 2.3. Cortical Auditory Evoked Potential (CAEP) Recording

Postoperative CAEP measurements were performed using the Fonix^®^ HEARLab System (Frye Electronics, Tigard, OR, USA) to assess late cortical responses. Stimuli consisted of three naturally spoken consonant–vowel tokens—/m/, /g/, and /t/—representing low-, mid-, and high-frequency speech sounds, respectively. Stimuli were delivered in the free field at 55 dB SPL through a loudspeaker positioned at a 1 m distance and 45° azimuth from the test ear. In CI users, the loudspeaker was oriented toward the implanted side. The orientation differed between groups because directing the stimulus toward the implanted ear is essential to ensure that cortical responses originate from the electrically stimulated auditory pathway. This prevents potential influence from the non-implanted or better-hearing side and follows standard pediatric CI CAEP recording procedures. Electrodes were placed at Cz (active), M1/M2, and Fpz (ground) according to the international 10–20 system, maintaining impedance below 5 kΩ [1]. Each child sat quietly on a parent’s lap or alone, watching a silent video to maintain calmness during the 25–35 min recording session. The HEARLab system automatically analyzed the cortical waveform, calculating a *p*-value for each speech sound to confirm the statistical presence of a true CAEP.

**Figure 1:**
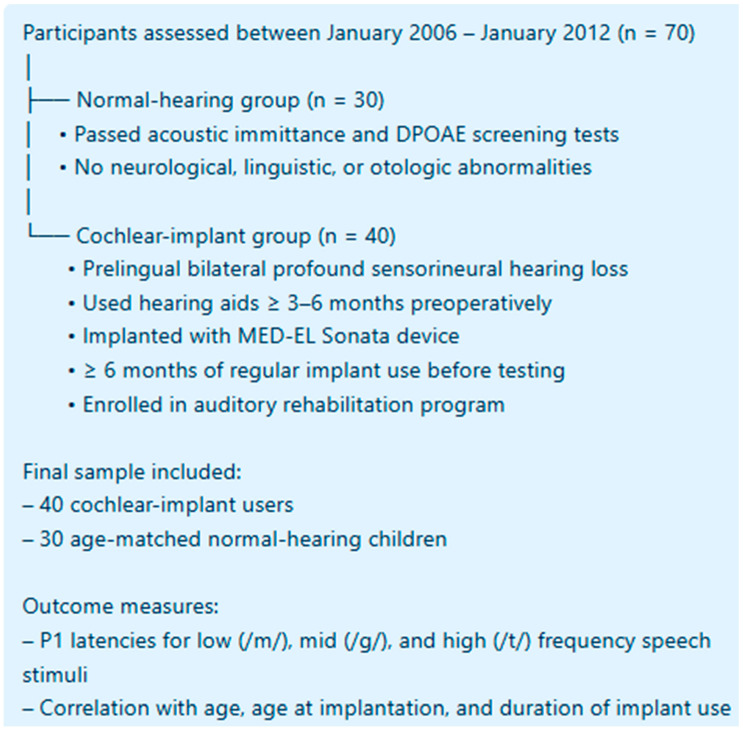
Flowchart of the Study Design.

The P1 component—the first prominent positive deflection occurring between 50 and 150 ms post-stimulus—was identified as the primary latency marker of cortical maturation. For clinical interpretability, normative latency intervals for each stimulus were derived from the normal-hearing cohort and used as internal reference values. The P1 response was recognized within the 50–300 ms window and defined as the earliest reproducible positive peak among the first three deflections following stimulus onset [1,12] (Figure 1).

### 2.4. Statistical Analysis

All statistical analyses were performed using SPSS version 26.0 (IBM Corp., Chicago, IL, USA). Descriptive data were expressed as mean ± standard deviation (SD). The Pearson correlation coefficient (r) was applied to evaluate the relationship between P1 latency and age in the normal-hearing group and between P1 latency and duration of implant use in the cochlear implant (CI) group. The strength of correlation was categorized as weak (|r| < 0.3), moderate (0.3 ≤ |r| < 0.6), or strong (|r| ≥ 0.6). A *p*-value < 0.05 was considered statistically significant for all analyses. A multivariate regression model (e.g., P1 latency = β_1_(age) + β_2_(age at implantation) + β_3_(duration of implant use)) could not be created because (i) age at implantation showed significant multicollinearity with implant-use duration and would yield unstable parameter estimates, and (ii) several key covariates—such as etiology of hearing loss, daily CI use hours, and structured rehabilitation metrics—were not consistently documented in the retrospective dataset. Due to these limitations, Pearson’s correlation analysis was used as the most statistically appropriate method for the available data.

## 3. Results

**Figure 2:**
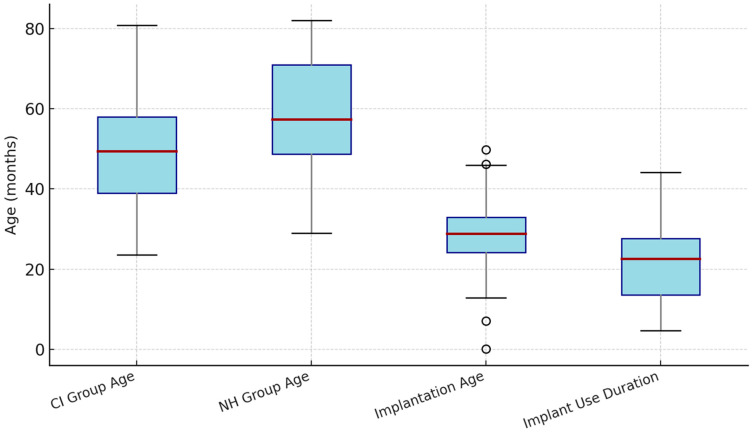
Distribution of chronological age, implantation age, and duration of implant use among cochlear-implant (CI) users and normal-hearing (NH) children. Boxes indicate interquartile ranges; whiskers show minimum–maximum values; red lines represent medians.

A total of 70 children were included in the study, consisting of 40 cochlear implant (CI) users (23 females, 17 males) and 30 normal-hearing (NH) children (17 females, 13 males). The mean age of the CI group was 53.23 ± 14.98 months (range: 24–82), and the mean age of the NH group was 58.93 ± 17.05 months (range: 15–84), with no statistically significant difference between groups (*p* = 0.180). The mean age at implantation for CI users was 28.83 ± 11.42 months (range: 12–72), and the mean duration of implant use was 22.38 ± 8.83 months (range: 12–40). Gender distribution was similar between the two groups (*p* = 0.940) (Table 1, Figure 2).

**Figure 3:**
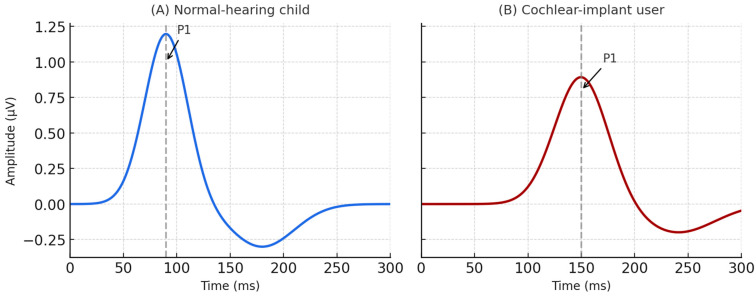
Examples of cortical auditory evoked potential (CAEP) waveforms recorded using the /m/ speech stimulus at 55 dB SPL. The P1 component (positive deflection between 50 and 150 ms) reflects cortical auditory response latency; compared with the normal-hearing child (**A**), the cochlear-implant user (**B**) demonstrates delayed and lower-amplitude cortical activation.

Cortical auditory evoked potentials (CAEPs) were successfully recorded in all participants using the /m/ speech stimulus at 55 dB SPL. In the normal-hearing group, the P1 component appeared as a distinct positive deflection occurring approximately between 80 and 100 ms after stimulus onset. In the cochlear-implant group, the P1 peak was observed at a later latency, approximately between 140 and 160 ms. The morphology of the waveform was clearly identifiable in both groups. The normal-hearing children exhibited earlier and higher-amplitude P1 responses, while cochlear-implant users showed delayed and lower-amplitude responses, consistent across all recordings (Figure 3).

**Figure 4:**
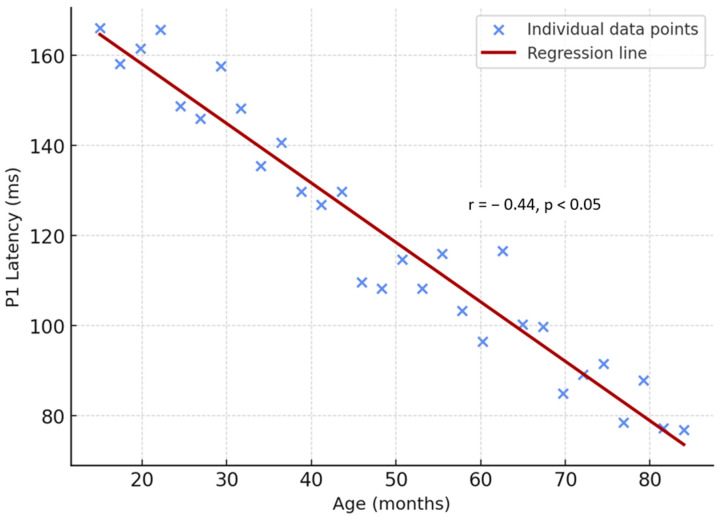
Correlation between chronological age and P1 latency in normal-hearing children. The inverse relationship (r = −0.44, *p* < 0.05) demonstrates that cortical auditory response latencies shorten with increasing age, consistent with normal auditory maturation.

The correlation analysis and corresponding distribution are shown in Figure 4. In the normal-hearing group, a negative correlation was observed between chronological age and P1 latency values for low-, mid-, and high-frequency speech stimuli (/m/, /g/, and /t/). Statistical analysis using the Pearson correlation test revealed an inverse relationship (r = −0.44, *p* < 0.05). As the age of the participants increased, P1 latencies measured across all frequency stimuli decreased (Figure 4). The correlation coefficients observed in both groups (r values between −0.37 and −0.49 for CI users and r = −0.44 for NH children) fall within the weak-to-moderate range, indicating modest effect sizes.

**Figure 5:**
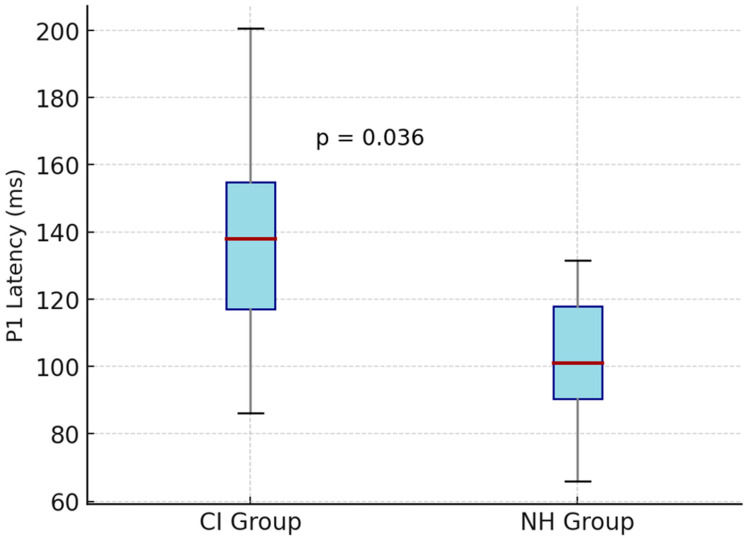
Comparison of P1 latencies obtained with the low-frequency /m/ stimulus between cochlear-implant (CI) and normal-hearing (NH) groups. The horizontal red line represents the median, and the box indicates the interquartile range (25th–75th percentiles). The whiskers extend to the minimum and maximum values. The CI group exhibited significantly longer P1 latency (*p* = 0.036).

**Figure 6:**
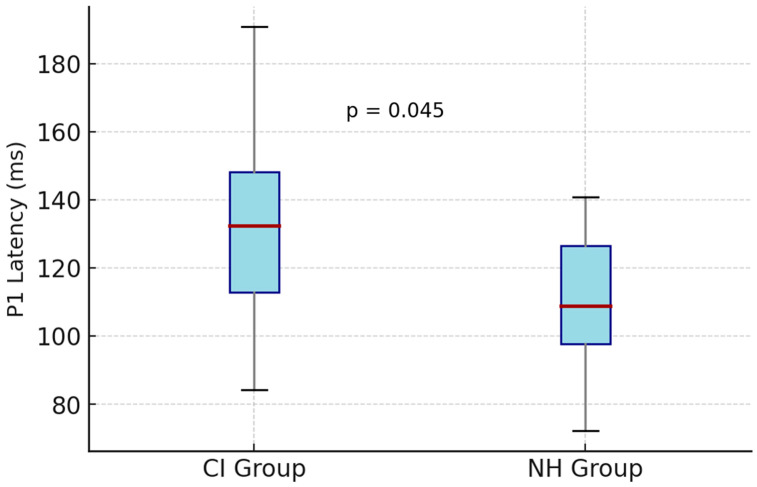
Comparison of P1 latencies obtained with the high-frequency /t/ stimulus between CI and NH groups (*p* = 0.045). The horizontal red line represents the median, and the box indicates the interquartile range (25th–75th percentiles). The whiskers extend to the minimum and maximum values. The CI group exhibited significantly delayed cortical responses.

The mean P1 latencies obtained for each speech stimulus were compared between the cochlear-implant (CI) and normal-hearing (NH) groups. For the low-frequency /m/ stimulus, the mean P1 latency was 145 ± 30 ms (range: 92–271) in the CI group and 103 ± 21 ms (range: 79–148) in the NH group (*p* = 0.036). For the mid-frequency /g/ stimulus, mean P1 latency values were 132 ± 27 ms (range: 70–225) for the CI group and 115 ± 25 ms (range: 71–193) for the NH group (*p* = 0.541). For the high-frequency /t/ stimulus, mean P1 latency was 139 ± 28 ms (range: 88–238) in the CI group and 111 ± 22 ms (range: 69–190) in the NH group (*p* = 0.045) (Table 2, Figure 5 and Figure 6).

Based on the normal-hearing cohort, frequency-specific normative P1 latency ranges were as follows: /m/ (low-frequency): 80–120 ms; /g/ (mid-frequency): 95–135 ms; and /t/ (high-frequency): 90–130 ms. These ranges align with published pediatric CAEP studies and provide a reference framework for evaluating latency deviations in CI users (Table S1).

**Figure 7:**
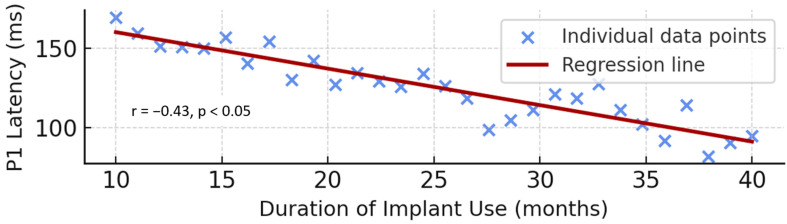
Correlation between duration of cochlear implant use and P1 latency. A significant negative correlation demonstrates that increasing implant use duration corresponds to reduced cortical response latencies (r = −0.43, *p* < 0.05).

The relationship between implant use duration and P1 latency is presented in Figure 7. In the cochlear-implant group, a negative correlation was found between the duration of implant use and P1 latency values for all frequency stimuli (/m/, /g/, and /t/). The correlation coefficients were r = −0.37 for /m/, r = −0.49 for /g/, and r = −0.43 for /t/ (*p* < 0.05 for all) (Figure 7).

## 4. Discussion

This study used speech-evoked cortical auditory evoked potentials (CAEPs) to evaluate cortical maturation in pediatric cochlear-implant (CI) users and age-matched normal-hearing peers. Across the three tested speech tokens (/m/, /g/, /t/), P1 latencies were longer in CI users for the low- (/m/) and high-frequency (/t/) stimuli but not for the mid-frequency (/g/) token. In normal-hearing children, P1 latency decreased with increasing age, while in the CI group longer implant-use duration was associated with shorter P1 latencies. These results confirm that P1 serves as a reliable biomarker of auditory cortical development under electrical hearing and highlight frequency-specific differences that may be clinically important for mapping and rehabilitation.

Visram et al. showed that aided CAEPs with brief, frequency-specific synthetic speech stimuli can be obtained with good sensitivity, repeatability, and feasibility in infants, underscoring their value as an objective complement to behavioral measures in early clinical pathways [1]. Recent clinical series emphasize CAEPs as an objective complement to behavioral measures for verifying aided audibility and documenting cortical access to speech cues, including in CI users [2,30]. The present data add to this body of work by showing clear P1 peaks in both groups with predictable latency differences. In our clinical cohort, CAEP findings occasionally contributed to mapping decisions. For example, delayed or borderline P1 responses led clinicians to increase stimulation levels or modify frequency allocation to optimize cortical access to specific speech cues. In a small number of cases, absent or markedly delayed CAEP responses prompted intensified auditory–verbal therapy referrals and closer follow-up to ensure adequate auditory development. Although detailed device-optimization logs were not uniformly available due to the retrospective design, these examples underline the practical clinical utility of CAEPs in guiding individualized management in pediatric CI users.

In normal-hearing children, the inverse association between age and P1 latency matches contemporary datasets describing age-related shortening of cortical latencies across childhood [3,34]. In CI users, “hearing age” (effective exposure under electrical hearing) is a key driver of normalization: longitudinal and cross-sectional work shows that consistent device use is associated with progressive P1 latency shortening, while reduced use is linked to persistently delayed responses [7,8,13,34]. Our finding of negative correlations between P1 latency and duration of implant use across /m/, /g/, and /t/ tokens is consistent with those reports and with studies tying objective cortical metrics to usage patterns and speech performance after implantation [12,13,16,20]. Although statistically significant, the observed correlations represent weak-to-moderate effect sizes, which limits the inferential strength of the findings. This underscores the need for larger-scale, adequately powered studies to better quantify the relationship between P1 latency and auditory maturation variables.

A more comprehensive analysis using multivariate regression would allow simultaneous evaluation of several factors known to influence auditory cortical maturation—such as age at implantation, etiology of hearing loss, daily device use, and rehabilitation characteristics. However, these variables were not uniformly documented in this retrospective dataset, limiting the feasibility of multivariate modeling. Prospective studies with complete datasets are needed to determine the independent contribution of these factors.

Another relevant factor influencing cortical maturation is the etiology of hearing loss, as congenital, genetic, infectious, or perinatal causes may affect auditory pathway development differently. Because etiologic records were incomplete for several participants, we were unable to incorporate this variable into the statistical analysis. Future prospective studies with comprehensive etiologic profiling are needed to examine its independent contribution to P1 latency.

Between-group contrasts showed significantly prolonged P1 for /m/ (low) and /t/ (high) in the CI group, with no difference for /g/ (mid). Comparable latency delays in CI users have been reported across various paradigms—acoustic CAEPs, eCAEPs, and mixed designs—reflecting slower cortical processing under electrical input early in the rehabilitation course [2,12]. Frequency- or stimulus-specific heterogeneity has been noted in pediatric cohorts, where maturation rates and audibility profiles can differ across spectral regions, device programs, or mapping strategies [30,32,33]. The loudspeaker was oriented toward the implanted ear in CI users to guarantee direct stimulation of the target auditory pathway. This procedural adaptation is commonly used in pediatric CI CAEP protocols to avoid contralateral auditory input influencing cortical responses. While our mid-frequency token showed no between-group difference, the global negative association between P1 and device-use duration suggests that continued stimulation may further reduce latencies across the spectrum over time, as seen in longitudinal work [3,7,13].

Beyond scalp CAEPs, implant-centric techniques are maturing. Saravanan et al. demonstrated pediatric electrically evoked late-latency responses (eLLR/eCAEPs) using single-electrode stimulation at apical, middle, and basal cochlear regions and reported relationships with speech perception, supporting the construct validity of electrically evoked cortical timing measures [12]. Extending this, Bell-Souder et al. validated direct recording of eCAEPs through the CI system itself—without surface EEG—highlighting a pathway to integrate cortical physiology into fitting workflows and longitudinal monitoring [15]. The present scalp-recorded trends (delayed P1 early, shortening with experience) are consistent with these implant-based readouts, together indicating a convergent physiological signature of cortical engagement that could inform programming and counseling.

Emerging methods record cortical responses directly via the CI interface and show strong correspondence with scalp measures, supporting clinical translation of objective cortical metrics [15,20]. Our scalp-recorded trends—with delayed P1 early and shortening with experience—mirror these direct-to-implant findings and reinforce the role of cortical timing as a target for programming and counseling in pediatric CI care [12,15,20].

Several recent pediatric studies link shorter P1 latencies with better speech-language measures, and report continued improvements with sustained device use and optimized rehabilitation [3,7,8,16]. Although speech outcomes were not analyzed here, the observed latency-use relationships are directionally concordant with this literature, suggesting that the electrophysiological changes captured by P1 may reflect clinically meaningful gains that warrant prospective follow-up in our cohort [3,8,9,16].

Taken together, the results support embedding speech-evoked CAEPs into peri-operative protocols to (i) verify cortical access to frequency-specific speech cues, (ii) track neural maturation as a function of device exposure, and (iii) identify atypical trajectories warranting remediation (e.g., mapping review, targeted therapy) in line with recommended procedures [1,6]. Future work should prospectively pair CAEPs with continuous device-use metrics and standardized speech-language outcomes in order to test whether token-specific P1 changes forecast functional gains, as suggested by Franlund et al. [3]. A hybrid approach that combines scalp CAEPs with eLLR/eCAEPs may sharpen individual-level decision-making and accelerate responsive programming [3,4]. As a proof-of-concept study, the present work provides an initial framework for using speech-evoked CAEPs to evaluate auditory cortical maturation in pediatric CI users. A prospective, multicenter design integrating longitudinal CAEP assessments and standardized language outcomes would represent a logical and essential continuation of this research.

Compared with previously published studies, the present work adds several methodological and analytical distinctions. Visram et al. focused on the feasibility of aided CAEPs in infants [1], and Franlund et al. linked P1 latency to language outcomes in CI children [3]; however, both examined single-stimulus paradigms. In contrast, this study used three frequency-specific speech tokens (/m/, /g/, /t/) to provide a frequency-resolved view of cortical timing. Unlike Saravanan et al. and Bell-Souder et al., who assessed electrically evoked cortical responses through the implant interface [12,15], we analyzed scalp-recorded speech-evoked CAEPs in a clinically accessible setting. Our inclusion of an age-matched normal-hearing control group and correlation of P1 latency with implant-use duration for each token offers a more comprehensive, experience-dependent model of cortical maturation under electrical hearing. The selection of normal-hearing controls may appear unusual in a retrospective design; however, CAEPs were performed as part of routine clinical evaluation in cases where behavioral assessments were inconclusive. Thus, the control data were pre-existing clinical recordings rather than research-motivated tests, supporting the retrospective case–control framework.

## 5. Limitations of the Study

This single-center retrospective design limits causal inference and sensitive-period modeling. Daily device-wear time, programming parameters, and detailed rehabilitation metrics were not analyzed, although these factors may modulate cortical timing. The sample size was relatively small, which reduces statistical power and may contribute to the weak-to-moderate correlation strengths observed; larger prospective cohorts are needed to validate these findings and improve generalizability. Several clinically relevant covariates—such as etiology of hearing loss, daily CI usage hours, and structured rehabilitation characteristics—were not fully available in the clinical archive. For this reason, multivariate regression analysis could not be performed, limiting the ability to statistically separate chronological age from hearing-age effects on P1 latency. Additionally, etiologic data were incomplete in some participants, preventing an examination of how specific etiologic categories may differentially influence auditory pathway development and cortical maturation. Future multicenter prospective studies are needed to validate the proof-of-concept findings of the present work and to examine how frequency-specific CAEP maturation relates to standardized language and functional auditory outcomes. Another limitation is that CAEP data for the normal-hearing control group were obtained as part of clinically indicated assessments rather than research-specific testing, which may introduce heterogeneity in the clinical indications and testing context. Future prospective studies with standardized assessment protocols, complete etiologic profiling, and fully captured clinical variables are needed to clarify the independent predictors of cortical auditory maturation in pediatric CI users. Because clinical mapping records were not systematically documented in the archive, only limited qualitative examples of CAEP-guided programming adjustments could be reported.

## 6. Conclusions

In conclusion, speech-evoked CAEPs provide a reliable and objective indicator of auditory cortical maturation in children with cochlear implants. The present study demonstrated that P1 latency shortens with increasing age in normal-hearing children and with longer implant-use duration in cochlear implant users, reflecting experience-dependent plasticity of the auditory cortex. Frequency-specific analysis revealed that latency differences were most pronounced for low- and high-frequency speech tokens, emphasizing the value of multi-frequency testing in clinical follow-up. These findings support the integration of CAEPs into peri-operative and rehabilitation protocols as an electrophysiological tool to monitor cortical development, optimize device programming, and guide individualized auditory training in pediatric cochlear implant care.

## Figures and Tables

**Table 1:** Demographic and clinical characteristics of the study groups.

Variable	CI Group (n = 40)	NH Group (n = 30)	*p*-Value
	n (%) or Mean ± SD [Range]		
Gender			0.940
Female	23 (57.5)	17 (56.6)	
Male	17 (42.5)	13 (43.3)	
Age, months	53.23 ± 14.98 (24–82)	58.93 ± 17.05 (15–84)	0.180
Age at implantation, months	28.83 ± 11.42 (12–72)	–	–
Duration of implant use, months	22.38 ± 8.83 (12–40)	–	–

**Table 2:** Comparison of P1 Latencies Between Cochlear-Implant (CI) and Normal-Hearing (NH) Groups.

Stimulus (Frequency Range)	CI Group	NH Group	*p*-Value
	P1 Latency (ms, Mean ± SD)		
/m/ (Low frequency)	145 ± 30 (92–271)	103 ± 21 (79–148)	0.036
/g/ (Mid frequency)	132 ± 27 (70–225)	115 ± 25 (71–193)	0.541
/t/ (High frequency)	139 ± 28 (88–238)	111 ± 22 (69–190)	0.045

## Data Availability

The data supporting the findings of this study are available from the corresponding author upon reasonable request. The data are not publicly available due to privacy or ethical restrictions.

## Supplementary Materials

The following supporting information can be downloaded at: https://www.mdpi.com/article/10.3390/children13020222/s1, Table S1: Summary of P1 Latency Measurements.
