# Supplementary material for: Speech-Evoked Cortical Auditory Potentials as Biomarkers of Auditory Maturation in Children with Cochlear Implants
Source: Children (Basel). 2026 Feb 4;13(2):222. doi: 10.3390/children13020222 (PMC12938934; doi:10.3390/children13020222)
Supplement: Supplementary file 1 [file children-13-00222-s001.zip › children-3992664-supplementary.pdf]

Supplementary Table S1. Summary of P1 Latency Measurements.

Frequency	Group	Minimum (ms)	Maximum (ms)	Mean (ms) ± SD
/m/	Normal hearing	79	148	103 ± 21
/m/	Cochlear implant	92	271	145 ± 30
/g/	Normal hearing	71	193	115 ± 25
/g/	Cochlear implant	70	225	132 ± 27
/t/	Normal hearing	69	190	111 ± 22
/t/	Cochlear implant	88	238	139 ± 28
